# Computational Modeling of Inhibitory Transsynaptic Signaling in Hippocampal and Cortical Neurons Expressing Intrabodies Against Gephyrin

**DOI:** 10.3389/fncel.2020.00173

**Published:** 2020-06-16

**Authors:** Carmen A. Lupascu, Annunziato Morabito, Federica Ruggeri, Chiara Parisi, Domenico Pimpinella, Rocco Pizzarelli, Giovanni Meli, Silvia Marinelli, Enrico Cherubini, Antonino Cattaneo, Michele Migliore

**Affiliations:** ^1^National Research Council, Institute of Biophysics, Palermo, Italy; ^2^European Brain Research Institute, Rome, Italy

**Keywords:** gephyrin, intrabodies, computational model, hippocampus, transsynaptic signaling

## Abstract

GABAergic transmission regulates neuronal excitability, dendritic integration of synaptic signals and oscillatory activity, thought to be involved in high cognitive functions. By anchoring synaptic receptors just opposite to release sites, the scaffold protein gephyrin plays a key role in these tasks. In addition, by regulating GABA_A_ receptor trafficking, gephyrin contributes to maintain, at the network level, an appropriate balance between Excitation (E) and Inhibition (I), crucial for information processing. An E/I imbalance leads to neuropsychiatric disorders such as epilepsy, schizophrenia and autism. In this article, we exploit a previously published computational method to fit spontaneous synaptic events, using a simplified model of the subcellular pathways involving gephyrin at inhibitory synapses. The model was used to analyze experimental data recorded under different conditions, with the main goal to gain insights on the possible consequences of gephyrin block on IPSCs. The same approach can be useful, in general, to analyze experiments designed to block a single protein. The results suggested possible ways to correlate the changes observed in the amplitude and time course of individual events recorded after different experimental protocols with the changes that may occur in the main subcellular pathways involved in gephyrin-dependent transsynaptic signaling.

## Introduction

Scaffold proteins, key components of postsynaptic densities, play a crucial role in regulating synaptic transmission. They interact with the cytoskeleton to anchor postsynaptic receptors just opposite to presynaptic release sites. In addition, they regulate receptor trafficking in and out of postsynaptic sites (Kneussel and Loebrich, 2007; Choquet and Triller, 2013; Petrini and Barberis, 2014). The complex interplay among all these proteins crucially determines synaptic transmission and, at the same time, makes it extremely difficult to understand the role of each component, and the functional consequences of its malfunction, in the context of the synaptic protein-protein interaction network. Experimental techniques allowing to gain some insight into this process include pharmacological applications or gene-based interference approaches (gene knock-out or RNA interference), but it is generally very difficult to interpret the results because of more or less unknown collateral effects, such as pharmacological non-specificity or compensatory changes in pathways that were not the direct target of the manipulation. Most importantly, gene-based knock-out or interference approaches target the nodes of the intracellular protein networks, and do not allow targeting selectively the edges of the protein interaction network. On the other hand, interference approaches based on the intracellular expression of intrabodies provide the opportunity to specifically target protein-protein interaction edges (Cattaneo and Chirichella, 2019). In all these cases, a computational model could be of great help in figuring out the consequences of a specific intracellular interference experimental protocol of synaptic transmission.

In comparison to excitatory synapses, the PSDs of inhibitory ones are localized mainly on dendritic shafts or on the cell bodies (Sheng and Kim, 2011). At inhibitory synapses, the scaffold molecule gephyrin anchors glycine and GABA_A_ receptors to the subsynaptic membrane in front of presynaptic release sites (Pizzarelli et al., 2019). Gephyrin is a 93-kDa tubulin-binding protein, originally purified in association with glycine receptors (Meyer et al., 1995), which plays a key role in anchoring glycine and GABA_A_ receptors to synaptic membranes, Tyagarajan and Fritschy (2014). Gephyrin’s structure comprises an N-terminal (G-domain) connected through a linker region (C-domain) to a C-terminal (E-domain) (Sola et al., 2001, 2004). To control GABAergic synapses formation and clustering, gephyrin interacts with several proteins, including neuroligin 2, collybistin and GABAA receptors (Tyagarajan and Fritschy, 2014). The original view that gephyrin *via* self-oligomerization forms hexagonal lattices which trap glycine and GABA_A_ receptors in the right place at postsynaptic sites by linking them to the cytoskeleton (Sola et al., 2004) has been recently questioned. According to Grünewald et al. (2018), in contrast to the lattice model, which assumes a gephyrin to glycine receptor β subunit stoichiometry of 1:1, this high receptor occupancy could be reached only if the E-domain dimerization within gephyrin clusters is incomplete, as suggested by recent data showing rather loose and irregular organization of receptor clusters (Specht et al., 2013) with numerous potentially unoccupied binding sites (Patrizio et al., 2017). Three dimensional and quantitative nanoscopic techniques based on single molecule imaging have allowed determining the subsynaptic distribution of gephyrin and receptor complexes at inhibitory postsynaptic densities (Specht et al., 2013; Crosby et al., 2019; Yang and Specht, 2019). Gephyrin plays a central role in synaptic transmission since it contributes to maintain, in particular brain areas, an appropriate balance between Excitation (E) and inhibition (I), crucial for the right operation of neuronal circuits (Pizzarelli and Cherubini, 2011; Xue et al., 2014; Chiu et al., 2019; Lourenço et al., 2020). An impairment of the E/I balance leads to neuropsychiatric disorders such as epilepsy, schizophrenia and autism (Penzes et al., 2013; Cellot and Cherubini, 2014; Nelson and Valakh, 2015; Antoine et al., 2019).

In this article, we exploit a previously published computational method to fit spontaneous synaptic events (Lupascu et al., 2016), using a simplified model of the subcellular pathways involving gephyrin at inhibitory synapses. The model was used to analyze experimental data, obtained by recording synaptic currents at hippocampal or cortical inhibitory synapses, after interfering with gephyrin with different strategies, either with gephyrin-selective intrabodies or with a dominant negative inhibitor of gephyrin. The main goal was to gain insights on the possible consequences of gephyrin block on IPSCs, and to develop a computational approach to optimize the information that can be gained from fitting the data to models that are necessarily oversimplified and with parameters that often cannot be appropriately constrained with experimental findings.

In previous reports (Marchionni et al., 2009; Varley et al., 2011), gephyrin-specific single chain antibody fragments (scFv-gephyrin) were used to disrupt gephyrin clusters and GABAergic signaling. ScFv-gephyrin contained a nuclear localization signal able to relocate gephyrin from the membrane to the nucleus. This led to a reduced accumulation of gephyrin at GABAergic synapses with consequent reduction in frequency and amplitude of spontaneous and miniature inhibitory postsynaptic currents (sIPSCs and mIPSCs).

Here, by analyzing experimental recordings of spontaneous events with a computational model, we have been able to correlate the amplitude and time course of individual events with the changes that may occur in the main subcellular pathways involved in gephyrin-dependent synaptic transmission and in the generation of the overall inhibitory current. The model suggests which pathway can be most affected by gephyrin block and how this can be reflected in the shape of the recorded signal.

## Materials and Methods

### Intrabodies

Two different formats to express intrabodies against gephyrin have been used: scFv-gephyrin with a nuclear localization signal (NLS) and scFv-gephyrin targeted to the cytoplasm. The technique for isolating scFv-gephyrin has been reported (Zacchi et al., 2008). Briefly, the Intracellular Antibodies Capture Technology (Visintin et al., 2002) was used to select a single chain antibody fragment (scFv) or intrabody against the linker C domain (aa 153–348) of gephyrin (Zacchi et al., 2008), a neutral epitope on the gephyrin molecule. This intrabody, in the cytosolic version (scFv-gephcyto), is expected not to interfere significantly with the function of gephyrin. The same scFv, fused to a nuclear localization signal (NLS) (scFv-gephNLS), was able to efficiently and selectively remove gephyrin from the synapse and abolish its interaction with glycine and GABA_A_ receptors.

### Lentivirus Production

scFv-gephcyto and scFv-gephNLS, fused to EGFP (scFv-gephcyto-EGFP and scFv-gephNLS-EGFP) were PCR amplified and subcloned into the *Xba*I site of pRRLSIN.cPPT.CMV.PGK-GFP. WPRE lentiviral transfer plasmid using the following primers: Fwd 5′-ATGACACTAGTaccATGGGCGCGCATGCCG ATATT-3′, Rev 5′-TTATCCTCTAGActaATCCAGGCCCAGCA GTGGGTT-3′. Lentiviral particles were produced by transient transfection of transfer plasmids along with packaging plasmids into 293T cells. Briefly, a total of 4 × 10^6^ cells were seeded in 15-cm tissue culture dishes 24 h before transfection. Cells were supplied with fresh DMEM medium 2 h prior to transfection. 25 μg of the lentiviral vector was mixed with 9.86 μg pMD2G, 12.5 μg pMDLg/pRRE, 7 μg pRSV-Rev. The solution was mixed with 2 M CaCl_2_ and adjusted to 1. 425 mL with water, then mixed with 1.425 mL of 2 × HEPES-buffered saline and added drop-wise directly to the cells. The medium was replaced after 16 h, and the vector-containing supernatants were harvested 48 and 72 h after transfection. After filtering through a 0.45-μm-pore-size filter, the supernatants were then spun at 26,000 × *g* for 2 h in a Beckman ultracentrifuge Optima L-90k. After centrifugation, the viral pellets were re-suspended in PBS and stored at −80°C.

All experiments were carried out in accordance with the European Community Council Directive of November 24, 1986 (86/609EEC) and were approved by the local authority veterinary service. Experiments were designed to minimize the number of animals used and their suffering.

### Mouse Cortical Neurons in Culture

Cortical neurons in culture were prepared from mouse embryos (C57BL/6J) at days E16–17. Cortices were isolated, freed of meninges washed and pelleted at 220 X g for 1 min. Tissue was incubated at 37°C for 30 min with 0.02% trypsin; then, DNase I (80 μg/ml) and trypsin inhibitor (0.52 mg/ml) were added. Digested tissues were mechanically dissociated and centrifuged at 220 × *g* for 10 min. Dissociated cells were counted and a total of 1.5 × 10^6^ cells were plated on 3.5 cm dishes with coverslips pre-coated with poly-l-lysine and cultured in neurobasal medium supplemented with B-27 and glutamax (Gibco, Thermo Fisher Scientific). After 2 days, half of the medium was changed every 3–4 days. At DIV 3-6, cultured cells were transduced with lentivirus expressing scFv-gephyrin cytoplasmic or scFv-gephyrin NLS. Equal numbers of viral particles of empty or scFv expressing lentiviruses were used for transduction of neurons at MOI 10, on the basis of viral copy number measured using the Lenti-X p24 Rapid Titer Kit (Clontech).

### Rat Hippocampal Neurons in Culture

Hippocampal neurons in culture were prepared as previously described (Andjus et al., 1997). Briefly, 2–4 days old (P2–P4) Wistar rats were decapitated after being anesthetized with an i.p. injection of urethane (2 mg/kg). Hippocampi were dissected free, sliced, and digested with trypsin, mechanically triturated, centrifuged twice at 40 × *g*, plated in Petri dishes, and cultured for up to 14 days. At 7 DIV, hippocampal neurons in culture were transfected with EGFP alone or co-transfected with EGFP plus scFv-gephyrin NLS, using the calcium phosphate transfection method.

### Electrophysiological Recordings

#### Cortical Neurons

The whole-cell configuration of the patch-clamp technique in voltage clamp mode was used to record miniature inhibitory postsynaptic currents (mIPSCs) from cortical neurons in culture (10 to 14 DIV), transduced with lentiviruses expressing scFv-gephyrin cytoplasmatic, scFv-gephyrin NLS with EGFP or EGFP alone. Cultured cells were maintained at room temperature (22−24°C) in artificial cerebrospinal fluid (ACSF) containing (in mM): NaCl 145, KCl 2, CaCl_2_ 2, MgCl_2_ 2, Glucose 10, Hepes 10 (pH 7.3, adjusted with KOH). Electrodes had a resistance of 4–5 MΩ when filled with an intracellular solution containing (in mM) KCl 150, CaCl_2_ 1, MgCl_2_ 2, EGTA 1, Hepes 10, Na_2_ATP 2 (pH 7.3, adjusted with KOH; 290 mOsm). mIPSCs were recorded in the presence of DNQX, D-APV and TTX (to block AMPA, NMDA receptors, sodium currents and propagated action potentials), from a holding potential of −60 mV, using a Multiclamp 700B amplifier (Axon CNS, Molecular Device). Series resistances were not compensated to maintain the highest possible signal-to noise and were monitored throughout the experiment. Cells exhibiting 15–20% changes in Rs were excluded from the analysis.

#### Hippocampal Neurons

Spontaneous GABA_A_-mediated postsynaptic currents (sIPSCs) were recorded from rat hippocampal neurons in culture transfected with scFv-gephyrin NLS associated with EGFP or transfected with EGFP alone. In some experiments cultured cells were transfected with the N-terminal truncated gephyrin polypeptide (amino acids 2–188) fused to EGFP.

Spontaneous IPSCs were obtained at room temperature (22−24°C) using a Multiclamp 700 A amplifier (Axon CNS, Molecular Device). Patch electrodes were pulled from borosilicate glass capillaries (Hilgenberg, Malsfeld, Germany). They had a resistance of 4–6 MΩ when filled with an intracellular solution containing (in mM): CsCl 137, CaCl_2_ 1, MgCl_2_ 2, 1,2-bis(2-aminophenoxy)ethane-N,N,N = N = -tetra-acetic acid (BAPTA) 11, ATP 2, and HEPES 10 (pH 7.3–7.4, adjusted with CsOH). The composition of the external solution was (in mM): NaCl 137, KCl 5, CaCl_2_ 2, MgCl_2_ 1, glucose 20, and HEPES 10, pH 7.4, with NaOH. sIPSCs were recorded from a holding potential of −70 mV, in the presence of DNQX and CGP 55845 to block AMPA and GABA_B_ receptors, respectively. The stability of the patch was checked by repetitively monitoring the input and series resistance during the experiments. Cells exhibiting 15–20% changes in Rs were excluded from the analysis.

#### Data Analysis

Data acquisition and analysis were performed using pClamp (Molecular Device), after digitization with an A/D converter (Digidata1440 A, Axon Instruments). Data were sampled at 10 kHz and filtered with a cutoff frequency of 2 kHz. Spontaneous and miniature events were analyzed with the Clampfit 10.1 software (Molecular Device). This program uses a detection algorithm based on a sliding template. The template did not induce any bias in the sampling of events because it was moved along the data trace one point at a time and was optimally scaled to fit the data at each position. The detection criterion was calculated from the template-scaling factor and from how closely the scaled template fitted the data.

For the purposes of the computational model the traces were divided in three groups: A, B and C. The A group included mIPSCs from mouse cortical cells expressing EGFP (controls), EGFP plus scFv-gephyrin cytoplasmatic or EGFP plus scFv-gephyrin NLS. The B group included sIPSCs from rat hippocampal cells transfected with EGFP (controls) or EGFP plus scFv-gephyrin NLS. The C group included sIPSCs from rat hippocampal cells transfected with the N-terminal truncated gephyrin polypeptide fused to EGFP (delta 2-188) or with EGFP alone.

### Computational Procedure

We carried out all simulations using an integrated NEURON (v7.4, Carnevale and Hines, 2006) and Python (v2.7, Hines et al., 2009) parallel code on different High Performance Computing (HPC) systems: JURECA (Juelich Supercomputing Center, Germany) (CINECA, Italy), Piz Daint (Swiss National Supercomputing Centre CSCS), and the Neuroscience Gateway (San Diego, United States, Sivagnanam et al., 2013). The fitting procedure was identical to that discussed in Lupascu et al. (2016). Briefly, individual well-defined spontaneous synaptic events were selected from continuous patch-clamp recordings, avoiding events with substantial overlapping (Hines and Carnevale, 1997). For the fitting procedure, the NEURON built-in PRAXIS principal axis method for minimizing a cost function was used. As a cost function, we used the classic root mean squared error (RMSE) between the time course and amplitude of the experimental and simulated currents. All the fitting parameters had the same weight on the cost function. The parallel implementation used the NEURON’s Parallel Context class with a bulletin board style. For all simulations, the system was implemented with a synapse targeting a single compartment (10 μm in diameter and length), with passive properties commonly used for CA1 pyramidal neurons (Cm = 1 μF/cm^2^, Rm = 28,000 Ω/cm^2^) and a resting potential set at the voltage clamp value used in the experiments (−60 or −70 mV). Note that for our simulations, carried out using a single-compartment model under perfect voltage- and space-clamp conditions, the passive properties do not affect the measured amplitude and time course of the current generated by a synaptic activation. Furthermore, in a previous article (Lupascu et al., 2016), we used the time to peak of the current to test if the fits obtained with our procedure were affected by alterations in the amplitude and time course of the synaptic current caused by inadequate voltage clamp of dendrites or filtering properties of the membrane. The results (see Figure 6 in Lupascu et al., 2016) suggested that all the fitted traces are from synaptic events elicited near the soma.

All model and simulation files can be downloaded from the ModelDB website (a.n. 182129), and from the model catalog available on the Collaboratory Portal of Human Brain Project (HBP)^1^.

The jupyter notebooks used to configure and run the jobs on different HPC systems can be accessed from the Brain Simulation Platform of the HBP^2^.

### The Model

**FIGURE 1:**
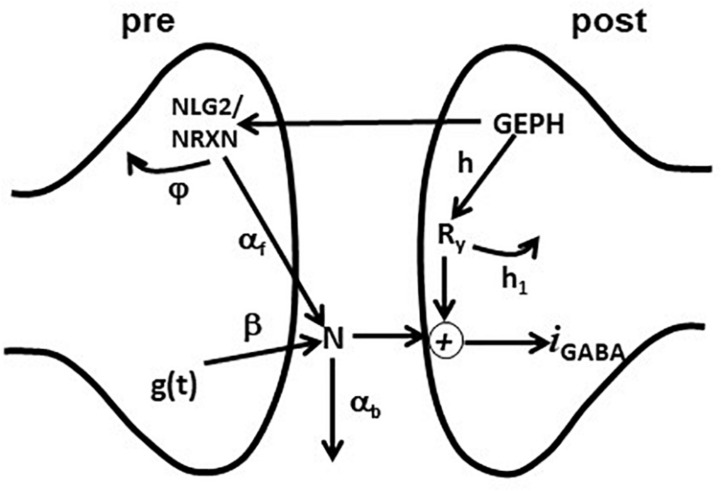
Schematic representation of the synaptic transmission model. The scheme represents the set of pre- and post-synaptic subcellular processes implemented in the model. See the main text for details.

In order to analyze the effects of scFv gephyrin cytoplasmatic or scFv-gephyrin NLS transfection, we used the kinetic model of synaptic transmission introduced and discussed in Lupascu et al. (2016), schematically illustrated in Figure 1.

The overall kinetic scheme was implemented as a perturbation of the classic double exponential function, widely used to model the experimentally observed amplitude and time course of a synaptic conductance, $g\,\left(t\right)=w\cdot \left[e\,x\,p\,\left(-\frac{t}{\tau_{d}}\right)-e\,x\,p\,\left(-\frac{t}{\tau_{r}}\right)\right]$, where we assumed that *w* is proportional to the amount of neurotransmitter released andτ*_*r*_*, τ*_*d*_* are the rise and decay time constant, respectively. This formulation implicitly takes into account the basic presynaptic mechanisms responsible for neurotransmitter release.

The model included an additional set of equations, modeling the effective modulation of the overall inhibitory current by subcellular pathways involving pre- and post-synaptic scaffolding proteins. For this purpose, we used the variables GEPH (gephyrin clusters), NLG2 (Neuroligin/Neurexin clusters), N (Neurotransmitter molecules), and Ry (Postsynaptic receptors), modeling their action through the following equations:

$$\frac{d\,\text{N}}{d\,t}=\beta \cdot \alpha_{f}\cdot g\,\left(t\right)\cdot \text{NLG}\,2-\alpha_{b}\cdot \text{N}$$

$$\frac{d\,\text{NLG}\,2}{d\,t}=\frac{\text{GEPH}}{1+\text{GEPH}/\left(2\cdot \text{NLG}\,2\right)}-\varphi \cdot \text{NLG}\,2$$

$$\frac{d\,{\text{R}}_{y}}{d\,t}=h\cdot \text{GEPH}-h_{1}\cdot {\text{R}}_{y}$$

After a synaptic activation, *g*(*t*) generates a number of neurotransmitter molecules, **N**, at a rate β. We have chosen this simplified effective implementation to take empirically into account all the mechanisms that can have a major role in determining the postsynaptic response (e.g., Liu, 2003). How they may regulate the amount of neurotransmitter released (in our case modeled by *w*) and its time course (modeled by τ*_*r*_*, τ*_*d*_*, and β) has been studied experimentally (e.g., Barberis et al., 2004, 2011). The effective number of neurotransmitter molecules available to the postsynaptic site is modulated by NLG2, which empirically models all the presynaptic gephyrin-dependent mechanisms acting on the neurotransmitter release; a portion of **N** is lost (e.g., neurotransmitter molecules diffuse away from the synaptic cleft) with a rate α_*b*_. The kinetics of **NLG**2 follows a Michaelis-Menten scheme, with a maximum value determined by **GEPH** and subjected to degradation with a rate constant φ. On the postsynaptic site, synaptic receptors **R_y_** are made available at a rate *h* and are removed at a rate *h*_*1*_. The synaptic current was calculated as:

$${\text{I}}_{\mathrm{GABAA}}=c_{1}\cdot \text{N}\cdot {\text{R}}_{y}\cdot \left(v-e_{r\,e\,v}\right)$$

where *c*_1_ is a constant, *v* the membrane potential and *e*_*rev*_ the reversal potential.

We used a simple formula not including detailed (and complex) pathways with additional variables and dynamics, because we considered it is sufficient to capture the overall effects of gephyrin on transsynaptic signaling, and because this implementation has important properties. For example, the variables *h* and *h*_*1*_ separate the effects caused by GEPH-dependent mechanisms on the post-synaptic response from the modulation caused by presynaptic pathways. Also, as we have previously shown (Lupascu et al., 2016), this implementation has the advantage that the set of differential equations can be solved analytically. The current *I*_*GABAA*_ can be described as:

$$I_{G\,A\,B\,A\,A}=I_{F\,A\,C\,T}$$

$$\frac{\left[\left(1-\alpha_{b}\,\tau_{d}\right)-\left(1-\alpha_{b}\,\tau_{r}\right)\right]\cdot {\text{e}}^{-\alpha_{b}\cdot t}+\left(1-\alpha_{b}\,\tau_{r}\right)\cdot {\text{e}}^{-\frac{t}{\tau_{d}}}-\left(1-\alpha_{b}\,\tau_{d}\right)\cdot {\text{e}}^{-\frac{t}{\tau_{r}}}}{\left(1-\alpha_{b}\,\tau_{d}\right)\cdot \left(1-\alpha_{b}\,\tau_{r}\right)}\,\left(v-e_{G\,A\,B\,A\,A}\right)$$

where $I_{F\,A\,C\,T}=c_{1}\cdot \frac{h}{h_{1}}\cdot \left[\frac{\left(2-\varphi \right)\cdot {\mathrm{GEPH}}^{2}}{2\cdot \varphi }\right]\cdot \beta \cdot \alpha_{f}\cdot w$

The equations above give a complete description of the effects of each pathway on the overall current. The amount of synaptic current generated by each synaptic activation will then be mainly dominated by the number of GEPH, squared, involved on the postsynaptic side, the variable φ, the hyperbolic dependence from the NLG2/NRXN turnover rateφ and the variable α_*b*_, the rate at which released neurotransmitter molecules diffuse away from the synaptic cleft α_*b*_.

A sensitivity analysis, testing the effects on *I*_*GABAA*_ amplitude and time constants has been carried out in Lupascu et al. (2016). Note that the choice to restrict our analysis to individual events allowed us to ignore short-term plasticity effects. We assumed that the average interval at which any given presynaptic cell generates an event was much longer than the current’s decay time.

## Results

We have designed a series of experiments to study how different ways of interfering with gephyrin function may affect in distinct ways spontaneous and action potential-independent transsynaptic GABAergic response and thus their modeling outcomes. Moreover, we used different brain areas, i.e., cerebral cortex and hippocampus, in order to investigate the generality of the findings. Indeed, in order to interfere with the activity of gephyrin, and to collect experimental data on inhibitory synaptic transmission, we exploited a well characterized intrabody recognizing the C-domain of gephyrin (Zacchi et al., 2008). Gephyrin C-domain is a linker between the NH2-terminal G-domain of gephyrin (which mediates gephyrin trimerization) and the COOH-terminal E-domain of gephyrin (responsible for the formation of the large networks of gephyrin beneath the synaptic membrane) (Sola et al., 2001, 2004). In the scFv-gephyrin cytoplasmic format, binding of the intrabody to this domain is not expected to have a strong inhibitory action *per se*, because this domain is not involved in significant protein−protein interactions. In the scFv-gephyrin NLS format, binding of the intrabody retargets gephyrin away from the synapse, into the nucleus, effectively depleting the synapses and lowering the concentration of gephyrin at synapses. We also used the (delta 2-188) truncated gephyrin polypeptide, comprising the N-terminal G-domain (amino acids 2-188) of gephyrin. This construct is a dominant negative inhibitor of gephyrin, inhibiting its trimerization.

Two modes of inhibition of gephyrin actions are therefore compared: (i) synaptic depletion of gephyrin (by the scFv-gephyrin NLS intrabody) and (ii) trimerization inhibition, with no change in total gephyrin concentration (by the delta 2-188 truncated gephyrin protein). The cytoplasmic construct is supposed to be the least disruptive treatment.

### Experiment A

Cortical neurons were virally transduced at DIV 7 and mIPSCs were recorded 4 days after (DIV 11) only from pyramidal neurons expressing EGFP. Miniature currents were reversibly blocked by bicuculline (10 mM) indicating that they were GABA_A_ receptor-mediated. The mean mIPSCs frequency was 1.5 ± 0.1 Hz in control (*n* = 35); 1.8 ± 0.2 Hz in the presence of scFv-gephyrin cytoplasmic (*n* = 24) and 1.1 ± 0.1 Hz in the presence of scFv-gephyrin NLS (*n* = 20). The mean mIPSCs amplitude was 53.2 ± 2.8 pA in control; 48.1 ± 3.5 pA in the presence of scFv-gephyrin cytoplasmic; 37.9 ± 1.7 pA in the presence of scFv-gephyrin NLS. No significant differences in frequency and amplitude were found between controls and scFv-gephyrin cytoplasmic (*p* = 0.37 and 0.19, respectively), but significant differences were found between controls and scFv-gephyrin NLS (*p* = 0.037 and 0.00028, respectively). These data show that scFv-gephyrin NLS effectively inhibits inhibitory synaptic transmission by removing gephyrin from the synapses and relocating it to the nucleus, where it is not active. The data also show that the scFv-gephyrin cytoplasmic intrabody, without a nuclear retargeting, does not alter GABAergic neurotransmission, confirming that the C-domain linker epitope recognized by the intrabody is not essential for gephyrin functions.

### Experiment B

In the hippocampus, spontaneous IPSCs were recorded from EGFP and EGFP plus scFv-gephyrin NLS transfected pyramidal neurons. Spontaneous events were recorded also from neighboring non-transfected pyramidal cells in the same dishes. No differences in amplitude, frequency and kinetics were observed between EGFP and non-transfected cells and therefore data were pooled together and considered as controls. Spontaneous events were reversibly blocked by bicuculline (10 μM) indicating that they were GABA_A_ receptor-mediated (*n* = 6). The mean sIPSCs frequency was 1.4 ± 0.1 Hz in controls (*n* = 10); 0.6 ± 0.2 Hz in the presence of scFv-gephyrin NLS (*n* = 7). The mean sIPSCs amplitude was 156.4 ± 30 pA in control; 75.8 ± 19.2 pA in the presence of scFv-gephyrin NLS. The differences were statistically significant (*p* = 0.005 and *p* = 0.036, respectively). These data, similar to those obtained in cortical neurons for mIPSC, strongly suggest that hampering gephyrin function by relocalizing the protein into the nucleus alters GABAergic neurotransmission.

### Experiment C

The effects of scFv-gephyrin NLS on sIPSCs from hippocampal neurons in culture were mimicked by the truncated gephyrin polypeptide comprising the N-terminal (amino acids 2-188) of gephyrin fused to EGFP (delta 1-188). In cells transfected with delta 2-188 fused to EGFP or with EGFP alone, the mean sIPSCs frequency was 0.91 ± 0.18 Hz in control (*n* = 10) and 0.42 ± 0.09 Hz in the presence of delta 2-188 (*n* = 9). The mean sIPSCs amplitude was 129.4 ± 18.8 pA in controls and 81.9 ± 13.2 pA in the presence of delta 2-188. These differences were statistically significant (*p* = 0.03 and *p* = 0.05, respectively).

**FIGURE 2:**
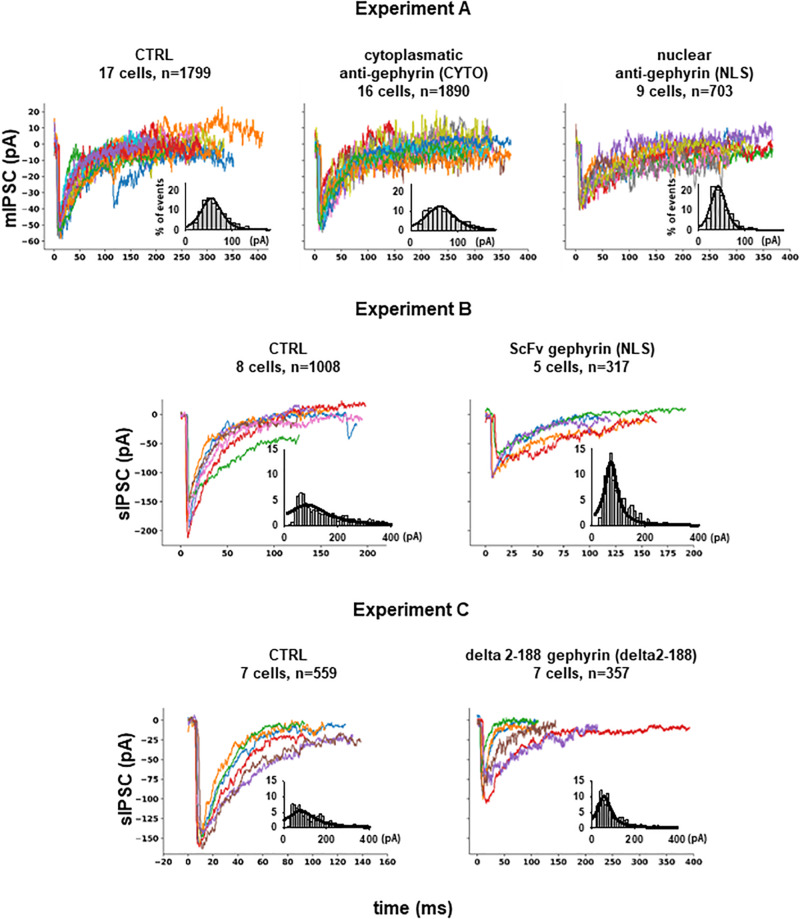
Inhibitory synaptic currents on hippocampal CA1 pyramidal neurons. Experiment **(A)** Representative raw experimental traces of miniature inhibitory post synaptic currents (mIPSCs) recorded from the three different experiments; the inset shows the distribution of the peak current from raw data (gray bar), and its fit using a Pseudo-Voigt, 4-parameter equation: $f=a\cdot \left(\frac{c}{1+{\left(\frac{x-x_{0}}{b}\right)}^{2}}+\left(1-c\right)\cdot e^{-0.5\cdot {\left(\frac{x-x_{0}}{b}\right)}^{2}}\right)$ with parameters: *a* = 0.1652, *b* = 23.0536, *c* = 0.4555, x_0_ = 54.5599 (*R* = 0.9768) for CTRL; *a* = 0.1239, *b* = 32.0002, *c* = 0.4609, x_0_ = 61.8422 (*R* = 0.9568) for CYTO and *a* = 0.2258, *b* = 16.5380, *c* = 0.3102, x_0_ = 42.5906 (*R* = 0.9638) for NLS. Experiment **(B)** Representative experimental traces of independent spontaneous inhibitory post synaptic currents (sIPSCs) recorded from EGFP and EGFP plus scFv-gephyrin NLS transfected neurons and distribution of peak sIPSCs (gray bar) from raw data; fitting parameters for the distribution: *a* = 0.0409, *b* = 90.5464, *c* = 1.0000, x_0_ = 84.2955 (*R* = 0.8364) for CTRL and *a* = 0.1252, *b* = 27.7427, *c* = 1.0000, x_0_ = 70.6842 (*R* = 0.9524) for NLS. Experiment **(C)** Representative raw experimental traces of independent spontaneous inhibitory post synaptic currents (sIPSCs) recorded from cells transfected with delta 2-188 fused to EGFP or with EGFP alone; fitting parameters for the distribution: *a* = 0.0556, *b* = 76.2090, *c* = 1.0000, x_0_ = 79.6038 (*R* = 0.8487) for CTRL and *a* = 0.1022, *b* = 37.1999, *c* = 1.0000, x_0_ = 55.9272 (*R* = 0.9324) for delta2-188.

From *Experiment A*, 4392 mIPSCs events were selected: 1799 under control conditions (cells expressing only EGFP), 1890 under scFv-gephyrin cytoplasmatic, and 703 under scFv-gephyrin NLS blocking condition. From *group B*, we selected 1325 raw spontaneous IPSCs traces: 1008 under control conditions and 317 under scFv-gephyrin NLS blocking conditions. From *group C*, we obtained a total of 916 raw sIPSC recordings: 559 under control conditions and 357 under delta 2-188 blocking conditions. For all experiments, the distribution of peak currents was well approximated by a 4-parameter pseudo-Voigt distribution, and the corresponding fitted parameters are reported in the legend of Figure 2, where we plot several representative traces from each experimental condition.

### Initial Fit of Model Parameters and Trace Classification

To fit the model parameters, we minimized the RMSE between the time course of the experimental and simulated currents. In order to take into account the fit, an average RMSE lower than 10% of the peak current was chosen as a threshold. During this first attempt we chose not to use additional constraints for the parameters other than a minimum and a maximum value. We carried out the fit procedure using 100 different initial parameter values (uniformly randomized within a large, 5 orders of magnitude, range) and up to 3000 iteration steps for each run. Because of the degeneracy phenomenon, observed in several biological systems (Edelman and Gally, 2001) including the CA1 region of the hippocampus (Patrizio et al., 2017), we expected many combinations of parameters giving equally good result for any given event. We found 290148 combinations of parameters resulting in a good fit for 6138 experimental events, 3926 events from group A (89.39% of the total number of raw traces), 1312 events from group B (99.02% of the total), and 900 events from group C (98.25% of the total). However, an analysis of these results showed no statistically significant differences between events recorded under control or after transfection. This was caused by the very large variability in both the properties of the experimental traces and in the range of parameter values fitting any given event.

We nevertheless performed a classification task using the ten model parameters calculated from the best fit of each trace, to test if it would be possible to classify individual traces as belonging to one of the groups. The Matlab Classification Learner app was used to perform the classification of the traces. Classification models were trained on 90% of the data and the resulting models were validated on the remaining part of the data using 10-fold cross-validation. The split was chosen randomly, but in such a way that after 10 repeats all samples have been left out once. Several methods were tested. In Table 1, we report the result for the method resulting into the best classification accuracy for each case.

**TABLE 1:** Accuracy of the models trained to classify the experimental traces.

**Class1**	**Class2**	**Class3**	**Accuracy**	**Classifier**
Group A − CTRL	Group A − CYTO	Group A − NLS	50.2%	Medium KNN
Group B − CTRL	Group B − NLS	−	77.7%	SVM (Quadratic)
Group C − CTRL	Group C − delta2-188	−	77.0%	SVM (Quadratic)

*The Matlab Classification Learner app was used to classify the experimental traces, using the model parameters as features. 10-fold cross-validation was applied. In the table we report the best accuracy score, which estimates a model’s performance on new data compared to the training data.*

These results show that, despite the large variability, in most cases the traces can be correctly classified as belonging to the correct group, with an accuracy well above chance level. This shows that while individual fitted parameters do not allow distinguishing the different datasets, their combination allows to do so. However, although the correct classification of a trace as belonging to a specific group is a valuable information, it cannot give any insight into how the expression of the different interference constructs can alter the synaptic transmission process. We then performed a more detailed analysis.

### Analysis of Each Experiment

The lack of a statistical difference, between model parameters that fit traces recorded under control or experimental conditions (transfection and viral transduction with the various constructs), is a clear indication that the fit procedure should be carried out after imposing more specific, physiologically plausible, constraints. For this purpose, from each experimental group, we selected only those events with a peak amplitude statistically consistent with the corresponding experimental average and standard deviation. The final number of events that were selected for fitting are reported in Table 2, and the average values obtained for control conditions are reported in Table 3.

**TABLE 2:** Number of traces used for the fit procedure and statistically consistent with the experimental distribution of the peak amplitude measured in the experiments.

	**CTRL**	**ScFv-gephyrin cytoplasmatic**	**ScFv-gephyrin NLS/delta2-188**
Exp. A	290	272	70
Exp. B	267	−	203
Exp. C	144	−	112

**TABLE 3:** Mean and standard deviation of the optimized parameters for control conditions.

	**Exp. A**	**Exp. B**	**Exp. C**
h	0.01930.0152	0.01940.0142	0.01680.0116
h_1_	0.12790.0754	0.09880.0633	0.12200.0801
α_f_	0.02460.0179	0.02610.0202	0.02920.0206
α_b_	1.5870e−052.2676e−05	9.6953e−061.7484e−05	1.4038e−052.0468e−05
β	56.226834.3897	59.242244.8992	58.821144.3356
τ_d_	55.930724.5969	28.456711.7380	29.718013.9137
τ_**r**_	0.79410.4377	0.74980.5063	1.50151.0914
ϕ	0.34770.1967	0.33450.2194	0.31910.1852
GEPH	2.26451.4180	2.80811.6634	2.68681.6748
*w*	8.7916e−046.4561e−04	9.0514e−046.8666e−04	0.00119.2808e−04

*The values correspond to those obtained for the best fit of each trace.*

To ensure that a change in any given parameter can be attributed to the transfected/transduced construct itself, rather than being a physiological fluctuation of control conditions, we analyzed in more details the results under control conditions for each experiment. The results are illustrated in Tables 4–6, where we report the relative difference in the parameters best fitting the traces recorded during each day of each experiment.

**TABLE 4:** Differences in the median between parameters in control conditions for Exp. A.

Exp. A day 1	τ_r_ +69.2%	τ_*r*_ +33.6%
Exp. A day 2		τ_*d*_ +21.0% τ_*r*_ −21.0%
	Exp. A day 2	Exp. A day 3
		

**TABLE 5:** Differences in the median between parameters in control conditions for Exp. B.

Exp. B day 1	α_f_ −25.1% α_b_ −0.06% τ_d_ +12.9%	β −23.8% τ_d_ +12.0% τ_*r*_ +89.2%
Exp. B day 2		h_1_ +44.9% β −28.4% τ_*r*_ +88.5% GEPH +30.3%
	Exp. B day 2	Exp. B day 3

**TABLE 6:** Differences between parameters in control conditions for Exp. C, calculated for the median; (N) indicate values calculated from the mean, for normally distributed values.

Exp. C day 1	τ_d_ −33.3% τ_*r*_ +121.9%	τ_d_ +67.0% (N) τ_*r*_ −44.4%	*w* −50.4%	τ_d_ + 4.7% (N) *w* −28.3%
Exp. C day 2		α_b_ −22.8% τ_d_ +163% τ_*r*_ −74.9%	τ_*r*_ −35.0%	h_1_ −33.6% α_b_ −22.8% τ_d_ +198.3% τ_*r*_ −57.8%
Exp. C day 3			τ_d_ −55.9% (N) τ_*r*_ +170.5% (N)	β +91.0% τ_*r*_ +68.4%
Exp. C day 4				τ_*d*_ +150.5% (N)
	Exp. C day 2	Exp. C day 3	Exp. C day 4	Exp. C day 5
				

As can be seen, within each experiment there were quite large fluctuations. This is reasonable, since it can be expected that synapses, in each neuron, can undergo large and independent changes during their entire life, according to the specific history of activity. For this reason, in comparing the results between control and after transfection or viral transduction, we did not consider any parameter showing a difference smaller than that observed under control conditions.

For each transfection/transduction experiment, we then carried out the fit after fixing the GEPH level to a value consistent with the experimentally measured change in the frequency of the spontaneous events, which can be expected to be proportional to the number of gephyrin molecules (Yu and De Blas, 2008).

#### Analysis of Experiment A

For the two cases of viral transduction in Experiment A, we considered the experimentally measured reduction by 26.6% in the mean mIPSCs frequency after scFv-gephyrin NLS, and no difference after scFv-gephyrin cytoplasmic. Since the fitting procedure for events under control conditions for Experiment A gave a mean GEPH values of 2.26, we carried out the fit procedure after fixing GEPH to this value for the events recorded in the presence of scFv-gephyrin cytoplasmic, and to 1.66 in the presence of scFv-gephyrin NLS. The average value for each parameter is reported in Table 7.

**TABLE 7:** Mean and standard deviation of the optimized parameters for each blocking condition.

**Exp. A**	**CYTO**	**NLS**
h	0.01670.0114	0.01710.0141
h_1_	0.12260.0841	0.13020.0780
α_f_	0.02270.0181	0.02690.0191
α_b_	1.8961e−052.3333e−05	1.6461e−052.2493e−05
β	49.947238.6689	57.232837.5945
τ_d_	52.908026.9647	72.304141.6816
τ_*r*_	0.72550.3790	0.89220.7532
ϕ	0.36210.2152	0.35390.2188
GEPH	2.2645	1.66063
*w*	7.2309e−046.6930e−04	6.7605e−045.2650e−04

*The values were calculated from those obtained for the best fit of each trace. Note that GEPH was fixed to a value consistent with the corresponding change observed experimentally in the mean mIPSCs frequency.*

**FIGURE 3:**
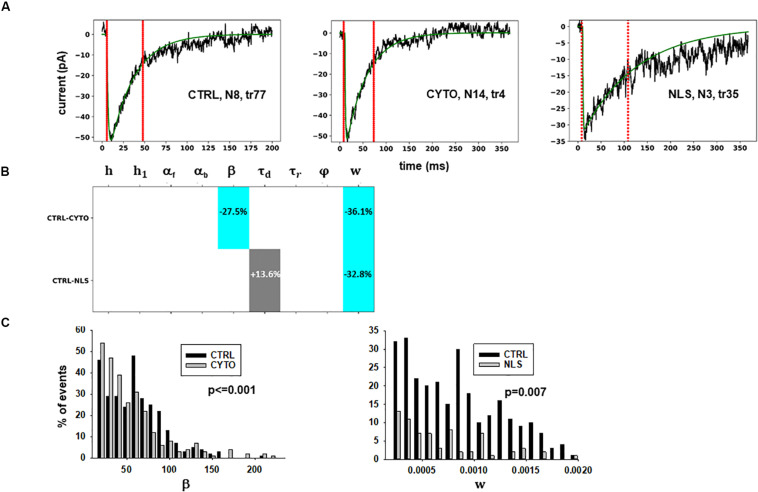
Analysis of traces from Experiment A. **(A)** typical best fits from events belonging to this group under control (left panel) and after transfection and viral transduction of different constructs (middle and right panels); the two vertical red lines highlight the portion of the trace used for the fit in each case, and the legend identifies the specific trace and cell; **(B)** Schematic representation of the difference between parameters. The colored boxes indicate cases for which *p* < 0.050: blue, the median under control is significantly lower than in the cells expressing the intrabodies; cyan, the median under control condition is significantly higher than after the transfection; gray, the change is within the range observed under control conditions. An empty box indicates no statistically significant difference; **(C)** distribution of values for β and *w* from all fits.

The results are illustrated in more details in Figure 3, where we report typical best fits (Figure 3A), a schematic representation of the differences between model parameters under different conditions (Figure 3B, values analyzed using a Pairwise Multiple Comparison Procedure, Dunn’s Method), and the distributions of two of the parameters for which our model predicted a statistically significant difference with respect to control (Figure 3C).

The results for traces obtained after scFv-gephyrin cytoplasmic transduction suggest that this protocol altered the release mechanism (related to β and *w*), whereas the model suggests that the transduction with scFv-gephyrin NLS may be more specific in affecting only the amount of neurotransmitter released (related to *w*). The results highlight the difference in the effects that can be generated by two different intracellular targeting (approaches) exploiting two distinct constructs, even using the same type of intrabody delivery (viral transduction).

#### Analysis of Experiment B

For Experiment B, considering the 57.1% reduction in the frequency of spontaneous events measured in the experiments in the presence of scFv-gephyrin, we carried out the fit for the traces recorded after transfection by fixing the GEPH value to 1.2 (from 2.8 under control conditions). The average values for each parameter are reported in Table 8.

**TABLE 8:** Mean and standard deviation of the optimized parameters for NSL blocking protocol.

**Exp. B**	**NLS**
h	0.01970.0134
h_1_	0.08260.0570
α_f_	0.03090.0234
α_b_	2.0499e−052.2877e−05
β	57.719634.8690
τ_d_	41.506021.2267
τ_*r*_	1.11790.8500
ϕ	0.30470.1892
GEPH	1.2035
*w*	8.5813e−046.4726e−04

*The values were calculated from those obtained for the best fit of each trace. Note that GEPH was fixed.*

**FIGURE 4:**
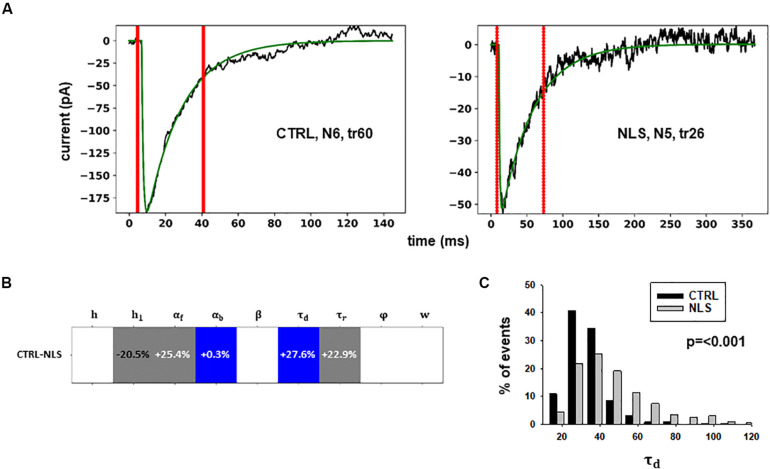
Analysis of traces from Experiment B. **(A)** typical best fits from events belonging to this group under control (*panel*) and after transfection and viral transduction (*right*); the two vertical red lines highlight the portion of the trace that used for the fit in each case; **(B)** Schematic representation of the difference between parameters. The colored boxes indicate cases for which *p* < 0.050: blue, the median under control is significantly lower than after the transfection; cyan, the median under control condition is significantly higher than after the transfection; gray, the change is within the range observed under control conditions. An empty box indicates no statistically significant difference; **(C)** distribution of values for τ_*d*_ from all fits.

Two typical best fits are reported in Figure 4A, a schematic representation of the differences between model parameters under different conditions in Figure 4B (values analyzed using a Pairwise Multiple Comparison Procedure, Dunn’s Method), and the distributions of two of the parameters for which our model predicted a statistically significant difference with respect to control in Figure 4C.

In this case, the model predicts presynaptic effects. The results suggest that blocking gephyrin with this protocol may cause a significant increase in the kinetic of neurotransmitter release (longer τ_*d*_), and a small but significant reduction in the amount of neurotransmitter diffusing away from the synaptic cleft (α_*b*_). This latter change is consistent with the experimental observation that scFv-gephyrin reduces the tonic inhibitory current (Marchionni et al., 2009). The results suggest that the changes in response to transfection with scFv-gephyrin NLS can be quite specific and involve the presynaptic side.

#### Analysis of Experiment C

**FIGURE 5:**
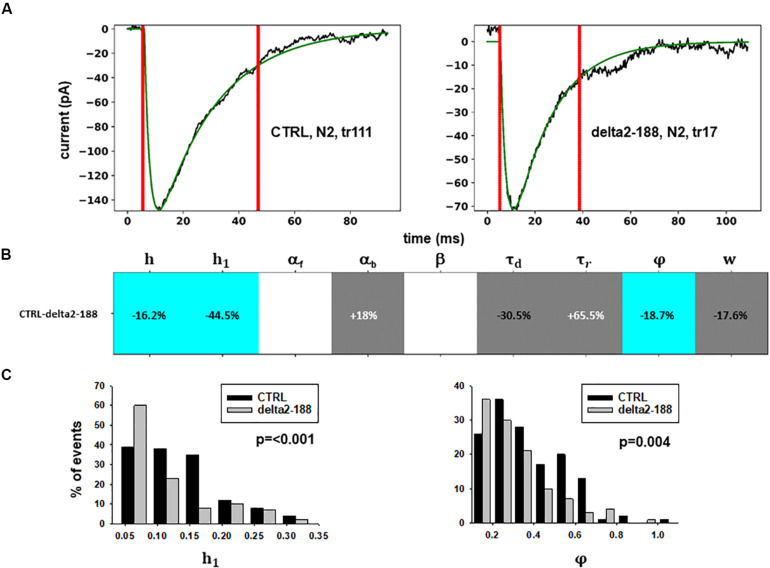
Analysis of traces from Experiment C. **(A)** typical best fits from events belonging to this group under control (*panel*) and after transfection and viral transduction (*right*); the two vertical red lines highlight the portion of the trace that used for the fit in each case; **(B)** Schematic representation of the difference between parameters. The colored boxes indicate cases for which *p* < 0.050: blue, the median under control is significantly lower than after the transfection; cyan, the median under control condition is significantly higher than after the transfection; gray, the change is within the range observed under control conditions. An empty box indicates no statistically significant difference; **(C)** distribution of values for *h*_*1*_and ϕfrom all fits.

For these experiments, we considered the 53.85% reduction in the frequency of spontaneous events measured in the presence of N-terminal truncated gephyrin polypeptides (delta2-188), and the fit for the traces recorded after transfection were carried out by fixing the GEPH value to 1.24 (from 2.7 under control conditions). Two typical best fits and a schematic representation of the differences between parameters under control and transfected conditions are shown in Figure 5. The average values obtained for the blocking conditions are reported in Table 9.

**TABLE 9:** Mean and standard deviation of the optimized parameters for each blocking condition.

**Exp. C**	**delta2-188**
h	0.01430.0112
h_1_	0.08870.0763
α_f_	0.03400.0229
α_b_	1.8283e−052.1808e−05
β	61.229839.2851
τ_d_	25.723616.1649
τ_*r*_	2.00821.2398
ϕ	0.25660.1672
GEPH	1.2401
*w*	9.1261e−048.3505e−04

*The values were calculated from those obtained for the best fit of each trace. Note that GEPH was fixed.*

In this experiment, the model indicates that delta2-188 expression can result in changes affecting both pre- and post-synaptic side, acting in opposite directions. For the post-synaptic side, the model suggests a reduction in the rate of receptor scaffolding (*h*); this would decrease the synaptic response. However, the reduction of the turnover rate of NLG2/NRXN proteins (φ)may lead to a possible compensatory change for the presynaptic side, since this increases the amount of the released neurotransmitter.

## Discussion

Several interesting considerations can be drawn from the analysis of the experimental recordings using the simple subcellular kinetic scheme of transsynaptic inhibitory signaling pathway proposed here. The most important, is that even the conceptually simplest action of blocking a single protein *in vivo*, can significantly alter the kinetics of many other biochemical pathways: gephyrin interference induces presynaptic/cleft changes including alterations of the kinetics of neurotransmitter release (β, *w*, and τ_*d*_) and/or neurotransmitter release away from the cleft. Should we have blocked presynaptic changes, the postsynaptic effects would have been stronger. This can be explained as a homeostatic mechanism in which the system tries to immediately react to maintain its physiological functionality. One particularly notable example of homeostatic modulation at the subcellular level is the experimentally observed paradoxical change in CaMKII phosphorylation, in response to manipulation of the extracellular calcium, to maintain a constant intracellular calcium concentration (Cohen and Fields, 2006).

The results suggest that changes occurring by blocking gephyrin may depend on the specific type of neuronal population at study and/or on the blocking protocol. Indeed, we find different changes in the parameters when removing gephyrin from the synapse (NLS construct) or when using the dominant negative delta 2-188 construct that inhibits gephyrin trimerization. In all cases, the model suggests that compensatory changes (i.e., working in a way which is opposite to what expected by the block of a given protein) may occur at both pre- and post-synaptic level. In particular, the NLS blocking protocol is the one less affecting all the other pathways, at least in cortical neurons, with changes restricted to the pre-synaptic location and limited to a reduction in the amount of neurotransmitter released. This may be a consequence of the fact that, for the mode of action of the NLS gephyrin intrabody, the constraint used (GEPH constant) forces the analysis on those changes that occur independently of the macroscopic depletion of gephyrin from the synapse. The delta2-188 protocol was instead the one most affecting synaptic transmission, generating significant changes in both pre- and post-synaptic pathways. Even for the case of the cytoplasmic intrabody, expected to be rather neutral with respect to gephyrin neutralization, such as scFv-gephyrin cytoplasmic, the model suggested statistically significant changes in the neurotransmitter release pathways.

The broad unifying context of the different experimental conditions compared in this article is related to the consideration of gephyrin in the context of the intracellular protein network. Intracellular protein networks are made of nodes and of edges connecting the nodes. Each protein is a node, connected by edges (protein−protein interactions) to other proteins. Protein hubs, such as gephyrin, are network nodes with a large number of edges. It is therefore clear that interfering with a node (such as what is achieved when analyzing the effects of gene knock-out) is very different than interfering with individual edges of a given protein, in the context of the network, and can lead to different results. This article combines different experimental ways to interfere with the gephyrin “node and edges” with the computational study of the resulting functional effects, measured quantitatively with electrophysiological techniques. In this respect the article provides a general methodological and conceptual advancement, and the approach described is general, even beyond the gephyrin case. As for gephyrin, by this cross-disciplinary approach we have learned in a formally stringent and novel way that “interfering with gephyrin impacts on both pre and postsynaptic “factors” including neurotransmitter release features and postsynaptic receptor clustering, reinforcing the intriguing idea that gephyrin can *trans*-synaptically organize the organization of inhibitory synapses.

Likewise, having performed experiments and computational analyses in different preparations, cortical and hippocampal neurons, provides an important validation of the overall approach, showing the generality of the findings.

In conclusion, the unifying view of the approach described in the article is that we have provided strong evidence for a new computational platform that will allow experimenters to investigate inhibitory synaptic transmission with their favorite inhibitors, drugs, or manipulations and learn how gephyrin and the protein network in which gephyrin is embedded regulate synaptic transmission in physiology and pathology.

The novelty of the analysis is therefore that it allows to quantitatively investigate the sensitivity of the network parameters in the presence of different modes of perturbation of the network.

All these effects point out to a general problem in analyzing experimental data on the manipulation of transsynaptic signaling pathways. Studies focusing on the effects caused by modulating a specific factor usually analyze one or at most a few possible related pathways; technical limitations obviously prevent to follow simultaneously many pathways, especially if they are not directly related to the modulation at study. This is a well-known (and accepted) problem for knock-out or other genetic manipulations. Our analysis has also shown that this problem may be even worse, if one considers that some of the parameters can significantly differ even among different cells in the same preparation under control conditions. This issue should be taken into account in analyzing experimental recordings, since it is part of the physiological variability caused by the individual and independent evolution of the synaptic network in each animal. A computational approach like the one that we have shown here, is a very convenient way to explore all these points.

The work presented is intended to highlight an approach, to solve the pitfalls and potential problems that arise when analyzing with a model experimental data on synaptic currents. The kinetic gephyrin model was highly simplified. The same approach could be readily applied to improved and more sophisticated models, to include, for instance, the spatial components of diffusion of the synaptic molecules involved (Choquet and Triller, 2013) and the oligomerization and aggregation of gephyrin (Ranft et al., 2017). On the experimental side, data could be obtained by equipping the gephyrin intrabodies with other effector functions, such as for instance suicide intrabodies (Melchionna and Cattaneo, 2007; Gross et al., 2016). From a more general methodological point of view, modeling the inhibitory transsynaptic signaling in the presence of gephyrin block may represent a “negative control model,” that helped better evaluating parameters important to model the physiological situation. This may become a standard procedure, to be followed when modeling neuronal and synaptic functions in physiological conditions, adding experimental constraints to better model parameters. To the best of our knowledge this is the first case using this approach.

Finally, to facilitate the community to follow our approach, we have created a set of public online use cases in the Brain Simulation Platform of the Human Brain Project^3^, implemented as interactive jupyter notebooks^2^. They allow users to analyze their own data, with the set of model kinetics we used in this work or with their own model, to test more specific or additional hypotheses. This can be done directly from a web browser, without the need to download or install any software or application, or without having a personal access to a supercomputer allocation.

## Data Availability Statement

The datasets generated for this study are available on the Knowledge Graph of the HBP at the following links: https://kg.ebrains.eu/search/instances/Dataset/31d100b53 522ba630499cc06ddecf6a6, https://kg.ebrains.eu/search/instances /Dataset/185144fef27b8ee7ed48c801f0cbb2b0, https://kg.ebrains. eu/search/instances/Dataset/bf19d2dceeb5aba0045d5d56a8e62 fc4, and https://kg.ebrains.eu/search/instances/Dataset/a59e3673 15cd7cbcfee42593201adc42. The model described in this study is available on ModelDB (a.n. 182129).

## Ethics Statement

The animal study was reviewed and approved by Minister of Health n.5/2015PR Protocol: Validation of recombinant antibodies against neuroligine and their interactive partner.

## Author Contributions

CL: substantial contributions to numerical simulation and data analysis. AM, FR, CP, DP, RP, SM, and EC: substantial contributions to the experiments. MM, CL, AC, EC, GM, and SM: substantial contributions to the conception and design of the work, to the analysis, and interpretation of data for the work. MM: drafting the work. All the authors revised the work critically for important intellectual content, approved the final version, and agreed to be accountable for all aspects of the work in ensuring that questions related to the accuracy or integrity of any part of the work are appropriately investigated and resolved.

## Conflict of Interest

The authors declare that the research was conducted in the absence of any commercial or financial relationships that could be construed as a potential conflict of interest. The reviewer AB declared a past co-authorship with one of the authors EC to the handling Editor.

## Footnotes

1 https://collab.humanbrainproject.eu/#/collab/1655/nav/75901?state=model.0a8b35ab-2b04-4421-9bb0-e54c5ca7546c

2 https://collab.humanbrainproject.eu/#/collab/1655/nav/66850

3 https://www.humanbrainproject.eu/en/brain-simulation/brain-simulation-platform/
